# Interaction between Workers during a Short Time Window Is Required for Bacterial Symbiont Transmission in *Acromyrmex* Leaf-Cutting Ants

**DOI:** 10.1371/journal.pone.0103269

**Published:** 2014-07-24

**Authors:** Sarah E. Marsh, Michael Poulsen, Adrián Pinto-Tomás, Cameron R. Currie

**Affiliations:** 1 Department of Bacteriology, University of Wisconsin, Madison, Wisconsin, United States of America; 2 Departamento de Bioquímica, Facultad de Medicina, Universidad de Costa Rica, San Pedro de Montes de Oca, San José, Costa Rica; 3 Centro de Investigación en Estructuras Microscópicas, Universidad de Costa Rica, San Pedro de Montes de Oca, San José, Costa Rica; 4 U.S. Department of Energy Great Lakes Bioenergy Research Center, University of Wisconsin, Madison, Wisconsin, United States of America; University of Sussex, United Kingdom

## Abstract

Stable associations between partners over time are critical for the evolution of mutualism. Hosts employ a variety of mechanisms to maintain specificity with bacterial associates. *Acromyrmex* leaf-cutting ants farm a fungal cultivar as their primary nutrient source. These ants also carry a *Pseudonocardia* Actinobacteria exosymbiont on their bodies that produces antifungal compounds that help inhibit specialized parasites of the ants' fungal garden. Major workers emerge from their pupal cases (eclose) symbiont-free, but exhibit visible Actinobacterial coverage within 14 days post-eclosion. Using subcolony experiments, we investigate exosymbiont transmission within *Acromyrmex* colonies. We found successful transmission to newly eclosed major workers fostered by major workers with visible Actinobacteria in all cases (100% acquiring, n = 19). In contrast, newly eclosed major workers reared without exosymbiont-carrying major workers did not acquire visible Actinobacteria (0% acquiring, n = 73). We further show that the majority of ants exposed to major workers with exosymbionts within 2 hours of eclosion acquired bacteria (60.7% acquiring, n = 28), while normal acquisition did not occur when exposure occurred later than 2 hours post-eclosion (0% acquiring, n = 18). Our findings show that transmission of exosymbionts to newly eclosed major workers occurs through interactions with exosymbiont-covered workers within a narrow time window after eclosion. This mode of transmission likely helps ensure the defensive function within colonies, as well as specificity and partner fidelity in the ant-bacterium association.

## Introduction

Symbiosis, the living together of unlike organisms [1], has been and remains critically important to the evolution of life on Earth. The success of symbiosis depends on the host's ability to properly acquire and maintain suitable symbionts. Microbial symbionts are either acquired vertically (from parent to offspring) or horizontally (from the environment or from individuals within the same generation, as can occur in the social insects). Highly constrained transmission allows few opportunities for partner switching. Symbiont transmission can be constrained by the need for physical interactions, temporal acquisition windows, and molecular specificity [2], [3]. Highly constrained transmission allows fewer opportunities for partner switching, increasing partner fidelity, which is important for maintaining stable interactions in specialized symbioses [4]. This type of evolutionary constraint can be imposed through several mechanisms, including recognition through partner-mediated molecular patterns by hosts and/or symbionts [2]. Establishing a symbiosis may rely on a critical bacterial acquisition window. For example, the alydid stinkbug, *Riptortus pedestris*, showed significantly greater acquisition of a horizontally transmitted *Burkholderia* midgut symbiont during the second instar larvae than other stages [3]. Rio and colleagues [5] have show similar importance of the timing of symbiont succession in the beneficial gut community of the European medical leech, *Hirudo verbana*. Limited symbiont acquisition periods likely function to increase colonization specificity [2] and are likely more common than currently recognized.

Fungus-growing ants are associated with fungal mutualists that they have been farming for approximately 50 million years [6]. The ants rely on their fungal cultivar as a food source, and in turn the fungus depends on the ants for collecting the substrate that serves as its nutrient source, for defence against microbial pests, and for propagation to new colonies [6], [7]. One defence employed by fungus-growing ants to protect their fungus garden is the maintenance of exosymbiotic Actinobacteria that produce antimicrobial compounds capable of inhibiting a specialized Ascomycete parasite (*Escovopsis* spp.) of the ants' cultivar fungus [8]–[14]. The ants appear to provide the Actinobacteria with nutrition from glands connected via channels to cuticular tubercles, which are present across most of the phylogenetic diversity of fungus-growing ants [15]. Two genera, *Atta* and *Sericomyrmex*, do not carry visible Actinobacteria [15], which has been suggested to be due to symbiont loss associated with a shift in relative importance of alternative defence mechanisms [16]. In species with Actinobacteria exosymbionts, virgin gynes carry a visible layer of exosymbiotic bacteria on their mating flights, suggesting vertical transmission of the exosymbiont between host-ant generations [10].

In *Acromyrmex* leaf-cutting ants, young major workers are present in the fungus garden. These ants typically exhibit abundant coverage of Actinobacteria, while minor workers and foraging (older) major workers carry little to no visible exosymbiont [17]. Major workers emerge from their pupal cases (eclose) symbiont-free and acquire the first visible traces of Actinobacteria detectable by electron microscopy 2–3 days post-eclosion, after which exosymbiont coverage increases for 10–15 days [18] (Figure S1). Major workers maintain high levels of symbiont coverage while they are present in the fungal garden, until approximately 25 days of age [18]. The subsequent reduction in coverage coincides with the time the ants begin to perform tasks outside the fungus garden and/or colony [18]. Culture dependent and independent methods, including elongation factor sequencing, suggests that each colony maintains a single strain of this symbiont, and that both *Acromyrmex echinatior* and *Acromyrmex octospinosus* hosted closely related actinobacteria in the genus *Pseudonocardia* [19]. Recent work, using pyrotag sequencing of a short segment of the 16 S ribosomal gene, supports that the population of exosymbiont on major worker cuticles in *A. echinatior* is dominated by a single phylotype of *Pseudonocardia*, and that colonies appear to maintain the same strain for many years [20]. This implies efficient transmission to newly eclosed major workers from an inoculation source, which Poulsen et al. [18] proposed could be major workers carrying abundant exosymbiont. Alternative sources, including the fungus garden, where low levels of Actinobacteria have been documented [21], seem unlikely, but have not been formally excluded. Other researchers have argued for low partner fidelity between fungus-growing ants and Actinobacteria, including individual colonies associating with multiple actinobacterial symbionts and readily acquiring new actinobacterial symbionts from the environment [22]–[25].

Here we explore the mode of Actinobacterial transmission in two species of *Acromyrmex* leaf-cutter ants, *A. echinatior* and *A. octospinosus*, using subcolonies with garden and leaf material. To determine if acquisition is a consequence of the presence of Actinobacteria-carrying major workers, we fostered newly-eclosed *Acromyrmex* major workers in the presence or absence of major workers carrying Actinobacteria, as well as with symbiont-free minor *Acromyrmex* workers, or symbiont-free *Atta* workers. We also fostered pupae with minor workers and a thorax dissected from a live major worker carrying a high level of exosymbiont coverage. Furthermore, to examine the time frame in which acquisition occurs, we reared newly-eclosed majors in subcolonies where Actinobacteria-carrying majors were introduced at different time points. Finally, specific conditions for within-colony transmission would help ensure long-term colony specificity in the ant-Actinobacteria association. To test for this, we isolated and sequenced 16 S rDNA of the Actinobacteria-symbiont *Pseudonocardia* from nine colonies, and compared these to sequences obtained when the same colonies were first collected 6–9 years earlier.

## Materials and Methods

### Ant colonies

The exosymbiont source and stability experiments were performed using the following colonies maintained at the University of Wisconsin-Madison: *Acromyrmex echinatior*: AP061104-01 Costa Rica; AeP5 & CC031209-02, CC031212-01 Panama; EC110523-1, EC110523-2 & EC110523-3 Costa Rica; *Acromyrmex hispidus fallax* UGM030327-02 Argentina; *Acromyrmex laticeps* UGM030330-04, CC030403-09, Argentina; *Acromyrmex niger* CC030327-02, Argentina; *Acromyrmex octospinosus* AL050505-11, CC031210-22, MP010908-1, SA010908-4, ST040116-01 & UGM020518-05 Panama; *Atta cephalotes*: AP061021-2 & AL050513-22 Costa Rica. Colonies were maintained at 24°C in the dark to mimic underground conditions, with overhead lights illuminated periodically for colony maintenance. Each ant colony was housed in a large outer plastic container (17.5 cm H×29 cm W×40 cm L) accommodating one smaller refuse dump container and one or two garden containers (ranging from 3.0 cm H by 7.5 cm L×7.5 cm W to 11 cm H×19.5 cm L×12.5 cm W, Pioneer Plastics). Each container had a 1 cm diameter hole drilled to allow ants to move in and out. Mineral oil was applied regularly to the top 4 cm of the outer container to prevent ants from escaping. Colonies were provisioned maple (*Acer* sp.) and oak (*Quercus* sp.) leaves (frozen in the winter and fresh during summer) three times per week. Leaves were supplemented with oatmeal, rice and cornmeal. Wet cotton balls were stored in the outer box to increase humidity.

Acquisition timing experiments were performed using *Acromyrmex octospinosus* colonies (SEM110828-1, RVC110826-1, RVC110813-3) collected in Heredia, Costa Rica in 2011. These colonies were maintained in the laboratory at the La Selva Biological station at 24°C under ambient lab light conditions. Each colony was housed in a larger plastic container (28 cm H×40 cm W×56 cm L) accommodating one smaller plastic container for refuse dump and one to three smaller plastic containers (ranging from 3.0 cm H by 7.5 cm L×7.5 cm W to 11 cm H×19.5 cm L×12.5 cm W, Pioneer Plastics) enclosing the colony fungus gardens. These colonies were provisioned with local flora, including leaves and flowers. Supplemental experiments were performed at the University of Wisconsin–Madison on the colonies mentioned above.

### Subcolony set-up: acquisition source

**Figure 1:**
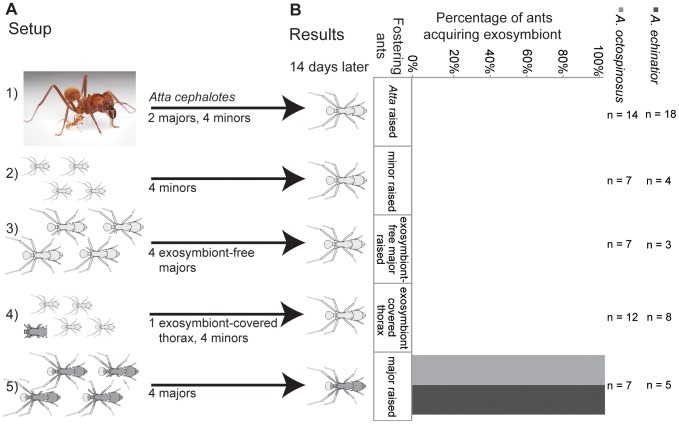
Acquisition source. a) Subcolony set-up. Newly eclosing *Acromyrmex* workers were fostered in subcolonies containing the following components 1) *Atta cephalotes* ants, a genus lacking visible symbiont, 2) minor workers (also lacking visible symbiont), 3) major workers without visible symbiont, 4) dissected thorax from major workers (changed every 48 hours) and minor workers (lacking visible symbiont) and 5) major workers with visible symbiont. Only pupae fostered with living major workers carrying *Pseudonocardia* developed symbiont coverage. b) The results of this experiment showed that *Acromyrmex echinatior* and *Ac*. *octospinosus* successfully acquired exosymbiotic bacteria only when pupae were raised in the presence of major workers carrying exosymbiont. Sample sizes (n) represent the number of focal ants surviving more than five days after eclosion. See Figure S1 for mortality data. Photo of *Atta cephalotes* used with permission, ©Alex Wild, Schematic ant drawings modified from Poulsen et al. [18].

We performed subcolony experiments to evaluate which colony components are necessary for successful exosymbiont transmission. Subcolonies were set up in small (4.0 cm H by 5.5 cm OD) clear plastic containers (Pioneer Plastics, Round Container 002C). After sterilizing the containers for at least 20 minutes using UV light, a Kimwipe moistened with distilled water was placed at the bottom to help provide humidity. A small (4.12 cm W×4.12 cm L×0.79 cm H) weigh boat (Fisher catalogue #08-732-112) was placed on top of the Kimwipe, and then the fungus garden, ants and pupa were added. A∼1 cm^2^ leaf fragment of pin oak (*Quercus palustris*) was added 24 hours or more after eclosion for the ants to cut and incorporate into the fungus garden. We started by establishing that *Acromyrmex* ants could acquire exosymbiont normally in a subcolony set-up consisting of a focal pupa, 0.1 g fungus, 2 major workers and 4 minor workers (n = 10 subcolonies) [26]. We monitored subcolonies daily to record eclosion date and visible exosymbiont coverage level for the focal ant until 14–21 days after eclosion, defining visible coverage as an indication of successful colonization. We performed environmental Scanning Electron Microscopy (eSEM) on a subset of the ants to confirm light microscopy findings (see Figure S1). Colony components were replaced as necessary (i.e., nurse ants in the case of death or fungal garden in the case of collapse). We explored which of the following colony component combinations would provide a source for exosymbiont transmission: 1) fungal garden, via cross-fostering with a related strain of fungus [a focal pupa, 0.1 g *Leucoagaricus* from *Atta cephalotes*, 2 major workers and 4 minor workers, n = 22 subcolonies; since pupae fail to eclose without assistance from older ants it was necessary to include ants in these set-ups]; 2) conspecific worker ants, via cross-fostering with another ant species with no visible Actinobacteria [a focal pupa, 0.1 g fungus garden, 2 major and 4 minor *Atta cephalotes* workers n = 61 subcolonies]; 3) minor workers [a focal pupa, 0.1 g fungus garden, 8 minor workers, n = 15 subcolonies], 4) major workers not carrying visible Actinobacteria [a focal pupa, 0.1 g fungus garden, 4 major workers cross-fostered with *Atta cephalotes* n = 12 subcolonies], 5) major workers carrying visible Actinobacteria [a focal pupa, 0.1 g fungus garden, 4 major workers, n = 17 subcolonies], and 6) minor workers with a thorax from a freshly dissected major worker with visible exosymbiont (changed every 48 hours to assure that bacteria were viable), to provide a bacterial source population [a focal pupa, 0.1 g fungus garden, 8 minor workers, and 1 exosymbiont-covered thorax, n = 23 subcolonies]. See Figure 1 and Table S1 for an outline of the experimental set-up and sample information by ant species. We classified workers by size, and then verified that all major ants carried visible exosymbiont, while minors did not.

### Subcolony set-up: acquisition timing

Preliminary cross-fostering experiments suggested that the period when callow workers could acquire exosymbiont was less than 24 hours (data not presented). This narrow temporal window emphasized the importance of delineating the time of eclosion. Here, the relevant feature of eclosion is likely to be the time when a callow worker can first interact with the environment (i.e., its cuticle is exposed to the environment outside the pupal case). Further, initial efforts to define a zero time point (i.e., the first moment an individual transitions from being a pupa to being an adult) revealed that the time it takes an individual to emerge from its pupal case is highly variable, ranging from minutes to hours. Attempts to visually verify the first holes in the pupal case via light microscopy lead to extremely high mortality. Given that pupae/callow workers often begin struggling to free themselves from their pupal case once this membrane is broken, as has been reported in fire ants [27], we defined the time of eclosion for experimental purposes as initiation of sustained kicking behaviour.

To determine the time after eclosion during which acquisition would predominantly occur, we fostered pupae with four minor workers with no visible exosymbiont from their natal colony. Subsequently, we added two majors with visible exosymbiont collected from the ants' natal garden at different time points after focal ant eclosion. Once these majors were added, they remained in the subcolony until the end of the experiment. We selected major workers whose Actinobacterial coverage and colony location made it likely that they were carrying abundant exosymbiont loads and were involved in brood care [18], [28]. Control colonies were set up with majors present initially, while experimental subcolonies were set up with majors added 0–1, 1–2, 3–4, 7–8 or 24–30 hours after focal ant eclosion. Leaf fragments were not added until 24 hours after eclosion to facilitate observations and to provide forage once majors capable of cutting were present.

### Long-term stability

A specific mode of within-colony transmission from older to younger major workers would suggest long-term stable association between individual ant colonies and their exosymbiotic bacterium. To test this we extracted and sequenced 16 S rDNA of the Actinobacteria symbiont *Pseudonocardia* from nine e-maintained colonies, and compared these to sequences obtained when colonies were first brought back to the from the field 6–9 years prior to this study. Other genera of Actinobacteria were not isolated at the time of colony collection, so we were unable to assess the stability of other potential members of the symbiont community. However, these are expected to occur in low frequency if at all [20]. At the time of colony collection, bacteria had been isolated from individual *Acromyrmex* ants by scraping visible *Pseudonocardia* from their propleural plates onto chitin agar plates, allowing bacteria to grow for 3 weeks, then transferring colonies with Actinobacteria-like morphology (small, white, dusty ball-like colony-forming units) to yeast malt extract plates [8], [19], [29]. We re-isolated *Pseudonocardia* using the same methods. Subsequently, we assessed whether ant colonies maintained the same bacterial symbiont by comparing the 16 S ribosomal RNA gene sequences of the original isolates with those obtained in this study. Bacterial DNA was extracted using a cetyltrimethylammonium bromide (CTAB) protocol described previously [8], [19], [29] and genomic DNA was quantified using a Nanodrop photospectrometer (Wilmington, DE, US) and diluted to 50 ng/ µl using TE. We amplified 16S rDNA using universal primers 27f (5'AGAGTTTGATCMTGGCTCAG'3) and 1492r (5'TACGGYTACCTTGTTACGACTT'3) [30]. Amplicons were sequenced on an ABI 3730xl DNA Analyzer at the University of Wisconsin Biotechnology Center (Madison, WI, US). We edited sequences using Sequencher 4.5 [31], did a preliminary alignment using ClustalX [32], edited the alignment using MacClade 4.07 [33], and used Mega 5.0 [34] to generate a Maximum likelihood phylogeny based on the Tamura-Nei model with all positions containing gaps and missing data eliminated.

## Results

### Source of exosymbiont transmission to newly eclosed workers

We explored the natural conditions under which newly eclosing *Acromyrmex echinatior* ants acquire Actinobacteria exosymbionts. Five of six focal pupae in control subcolonies (containing a focal pupa, fostering major and minor ants, and a leaf fragment) acquired exosymbiont normally, consistent with patterns of acquisition previously reported [18].

To test whether the ants' fungal garden could be the source of the *Pseudonocardia*, we conducted fungus-switching experiments. We fostered *Atta* and *Acromyrmex* pupae with majors and minors from their natal colonies, but with fungus garden fragments from a host ant of the other genera (e.g., *Atta cephalotes* ants and pupae with *Acromyrmex* garden). Switching of the fungal garden resulted in high mortality among callow workers (54% survival, n = 22 subcolonies). No *Acromyrmex echinatior* ants lived longer than five days post-eclosion (0% survival, n = 4 subcolonies), so all results reported here are for *Acromyrmex octospinosus* (58% survival, n = 12 subcolonies). None of the five *Atta* ants acquired exosymbiont when raised with *A. octospinosus* fungus garden (0% survival, n = 5 subcolonies). When *A. octospinosus* and *A. echinatior* pupae were cross-fostered with *Atta* garden and *Atta* workers (83% survival, n = 6 subcolonies), no majors acquired visible exosymbiont coverage (0% transmission, n = 5 subcolonies). These results indicate that the fungus garden is highly unlikely to be a significant source of exosymbiont inoculum.

No acquisition was observed when *Acromyrmex* ants were raised with their own fungal garden and *Atta* workers (n = 14 and n = 18 for *A. octospinosus* and *A. echinatior*, respectively), nor when pupae were raised with minors only (n = 7 and n = 4 for *A. octospinosus* and *A. echinatior*, respectively) or minors only but with the presence of an Actinobacterial source population (i.e., the thorax of a major worker with copious visible exosymbiont added to the fungal garden; n = 12 and n = 8 for *A. octospinosus* and *A. echinatior*, respectively). In contrast, all major worker pupae raised with *Acromyrmex* major workers and fungus from either *Acromyrmex* or *Atta* colonies acquired visible exosymbiont (100% transmission, n = 5, n = 7 and n = 7 for *A. octospinosus* all components, *A. octospinosus* ants/*Atta cephalotes fungus*, *A. echinatior* all components respectively). This indicates that major workers carrying visible levels of exosymbiont are necessary for normal acquisition. Further, our fostering of pupae in subcolonies with majors carrying no visible exosymbiont resulted in no workers acquiring exosymbionts (n = 7 and n = 3 for *A. octospinosus* and *A. echinatior*, respectively). These results thus show that direct contact with major workers carrying visible exosymbiont is necessary for normal exosymbiont transmission (Figure 1).

### Acquisition timing

**Figure 2:**
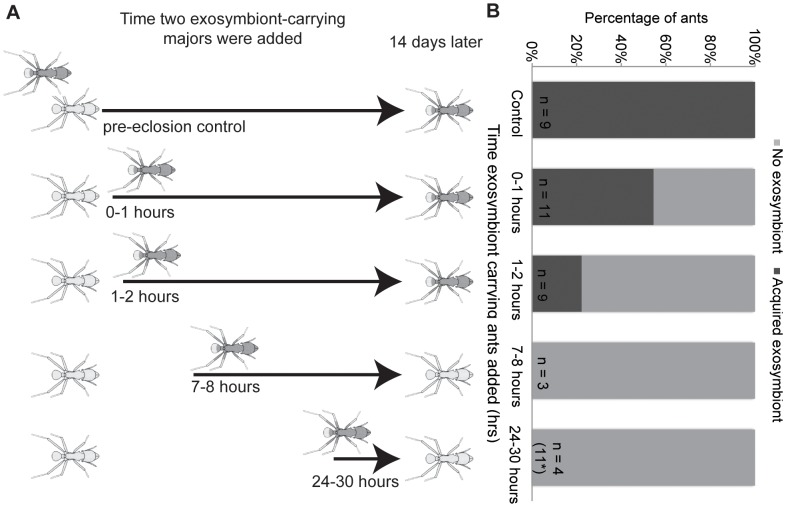
Acquisition timing. a) All set-ups contained one focal pupa, 4 minor workers and a piece of fungus garden. Major workers with visible exosymbiont were added at varying intervals from 0–1 hours to 24–30 hours post-eclosion. Controls contained major workers added at the time of subcolony set-up. Ants acquired in a gradient from 100% of controls to 0% of ants exposed after more than 2 hours. Drawings modified from Poulsen et al. 2003 and photos by S.E.M. b) Critical window period for exosymbiont acquisition in *Acromyrmex octospinosus*. The percentage of ants acquiring visible exosymbiont after exposure to exosymbiont-carrying majors introduced at varying times after eclosion. All pupae from 3–4 hour subcolonies died (n = 10), not shown. Sample sizes (n) represent the number of focal ants surviving more than five days after eclosion. See Figure S1 for mortality data. *An additional 11 subcolonies were set up in the lab due to high field mortality.

To explore whether exosymbiont acquisition is limited to a brief inoculation period, we allowed pupae to eclose without Actinobacteria-carrying majors present and then added Actinobacteria-carrying majors at different time intervals after major worker eclosion. Although mortality was high across all fostered pupae, a clear pattern in acquisition timing emerged (Figure 2). If exposure to major workers with visible coverage occurred 2 hours or longer after eclosion, these new major workers did not acquire Actinobacteria (Figure 2). In contrast, six of eleven ants exposed to workers with Actinobacteria in the 0–1 hour window after eclosion acquired exosymbiont, as did two of nine ants exposed in the 1–2 hour window. All control ants (100% transmission, n = 9 callow workers eclosing in the presence of Actinobacteria-carrying majors) acquired exosymbionts normally.

### Symbiont stability

**Figure 3:**
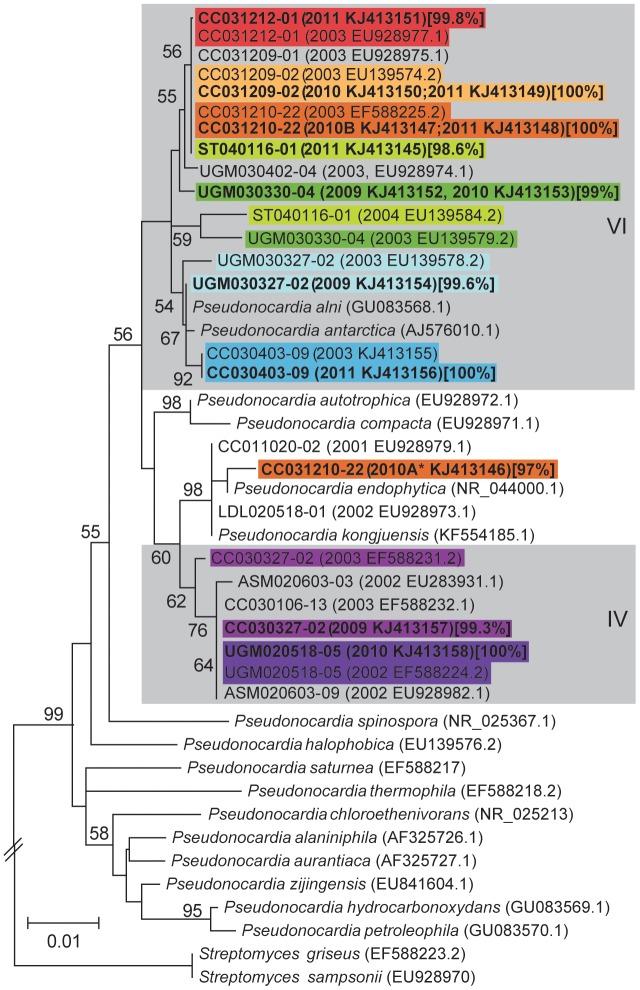
16S *Pseudonocardia* phylogenetic tree. A phylogeny showing bacterial sequences associated with individual leaf-cutting ant colonies over time and their free-living relatives (*italics*). The phylogenetic tree was generated by the maximum likelihood method based on the Tamura-Nei model and bootstrapped 1000 times. The scale bar represents the genetic distance between samples, reflecting the number of nucleotide changes per site. The tree was created from 16 S rDNA sequences of *Pseudonocardia* exosymbionts obtained from leaf-cutting ants at the time of colony collection (regular text) and up to 9 years later (bold text). Year(s) of isolation noted in parenthesis after the colony code. Identical sequences obtained at different times are represented by a single sequence with multiple dates. Clades of *Pseudonocardia* associated with leaf-cutting ants are labeled with roman numerals [8], [19] and depicted in grey boxes. Ant-isolates are labeled by colony number, followed by the year isolated and GenBank identification number in parenthesis. The percentage sequence identity with original isolates is given in brackets after all re-isolates. Isolations from the same colony are boxed in the same color to facilitate comparison. Note that bacteria isolated from all colonies remain in the same phylogenetic clade over time, with the exception of a different morphological type (*) isolated once from a colony with two other independent re-isolates identical to the original sequence isolated.

Transmission of symbionts from older to newly-eclosed majors within a narrow time window suggests long-term propagation of specific Actinobacteria symbionts within individual ant colonies. This predicted long-term serial transmission of specific symbionts within individual ant colonies was supported by re-isolations of *Pseudonocardia* from the same colonies over a 6–9 year period. Specifically, we isolated identical, or near identical (i.e., within 99.8% 16 S rDNA sequence identity) strains of *Pseudonocardia* symbionts from five colonies of *Acromyrmex* spp. ants following 8 years (four colonies: two *A. echinatior* CC031209-02 with two independent re-isolates and CC031212-01 with one independent re-isolate; *A. octospinosus* CC031210-22 with three independent re-isolates; and *A. laticeps* CC030403-09 with one independent re-isolate) and 9 years (one colony of *A. octospinosus* with UGM020518-05 with one independent re-isolate) of maintenance in the laboratory (Figure 3). Further, we isolated *Pseudonocardia* with >98.5% 16 S rDNA sequence identity, placing the strain in the same symbiont clade, from four colonies after 6–7 years of maintenance in the laboratory (ST040116-01, one independent re-isolate, 7 years; UGM030330-04, two independent re-isolates, 7 years; UGM030327-02, one independent re-isolate, 6 years; CC030327-02, one independent re-isolate, 6 years).

## Discussion

Many, if not all, metazoans depend on beneficial microbial symbionts. These microbes provide their hosts with a range of services including nutrition and defence. In a number of these host-symbiont associations, a high degree of specificity exists between host species and/or populations and individual species or specific phylogenetic clades of closely related strains of microbes [8], [35]–[37]. Specificity is expected to help ensure the presence of symbionts that confer appropriate benefits to the host [4], in addition to stabilizing the host-microbe association by preventing non-beneficial symbionts from competing with and potentially displacing beneficial symbionts [38]. Many hosts rely on vertical transmission from parents to offspring for between host-generation alignment of reproductive interest aiding partner fidelity [2]. In social hosts, the acquisition of symbionts is not only between host generations (new queens and colonies), but also between overlapping generations within individual nests. Our findings reveal a specific mechanism of within-colony transmission of cuticular Actinobacteria exists in two leaf-cutting ant species in the genus *Acromyrmex*, in addition to indicating that the opportunity for acquisition of symbionts by this mechanism is possible only during a narrow time window.

Older major workers had previously been proposed as the source of Actinobacterial transmission [39], so we experimentally tested this by exploring the conditions required for major *Acromyrmex* leaf-cutting ants to acquire natural populations of cuticular Actinobacteria. This confirmed that newly eclosed major workers could acquire exosymbionts in small subcolonies with leaf fragments, fungal garden, and adult majors and minors present. Subsequent experiments fostering (within genus) and cross-fostering (across genera) callow ants in simplified subcolonies (lacking particular components such as minor workers) allowed us to eliminate the possibility that fungus garden fragments, minors, and freshly dissected cuticle with Actinobacteria from majors could provide a source of inoculum of the bacteria. Our findings indicate that the presence of live Actinobacteria-carrying majors is necessary for successful transmission to occur, making this source the primary, likely sole, route of within-colony transmission for these two *Acromyrmex* species. Other fungus-growing ant genera carry Actinobacteria on the same locations of the cuticle as *Acromyrmex* [15], so this mode of within-colony transmission may be more general.

Our work indicates that successful exosymbiont transfer requires worker-to-worker contact occurring less than 2 hours after worker eclosion. The mechanisms responsible for this narrow time window for successful exosymbiont transfer are unknown, but are likely linked to the physiology of the ants, which actively maintain the bacteria on so-far unidentified cuticular secretions [15]. Our findings suggest that appropriate cuticular secretions may either only be available during a short time window or may change dramatically during the initial ontology of the ant host, potentially facilitating host-symbiont specificity by reducing the time where newly eclosed majors are susceptible to colonization and thereby potentially ‘vulnerable’ to invasion by undesired bacteria. The finding that successful establishment requires that callow majors are reared in the presence of live exosymbiont-carrying majors, indicates that the combination of the right conditions for Actinobacteria establishment include both cuticular receptiveness to colonization as well as direct major worker-to-callow major worker interactions during a brief critical time window. During this time period older majors interact closely with newly eclosed majors by antennating, walking on, and carrying them (S.E.M., 2009–2012, personal observations). Since major callow workers universally acquire Actinobacteria cover, the recognition of callow major eclosion, followed by behavioural interactions, by older workers must be highly efficient to assure efficient within-colony transmission. This limited acquisition period parallels findings in alydid stinkbugs, which can only be colonized by their *Buckholderia* gut-symbiont during a single phase of larval development [3].

A narrow time window for active transmission of specific Actinobacteria to newly eclosed major workers suggests that specific bacterial symbionts are continuously inoculated onto new individuals within ant colonies and therefore maintained over the lifetime of the colony. Andersen and colleagues [20] used 16 S pyrotag sequencing to show that individual colonies of *A. echinatior* from Panama are dominated by a single *Pseudonocardia* bacterium from either of two specific phylogenetic clades, consistent with previous findings [8], [19]. Further, the association of individual colonies with a bacterium from a specific clade appears to be stable over many years (reported up to17 years) [20]. To independently confirm this, and to provide more phylogenetic resolution by using near full-length 16 S rDNA sequences, we isolated *Pseudonocardia* exosymbionts from nine *Acromyrmex* colonies that had been maintained in the lab for up to 9 years. We found that 16 S DNA sequences were identical, or near identical, to those of the *Pseudonocardia* symbionts originally isolated from colonies at the time of collection. Five of these colonies were from the same Panamanian population as those in Andersen and colleagues [20], while the remaining colonies were from three other *Acromyrmex* species (*A. laticeps*, *A. hispidus fallax* and *A. niger*) collected in Argentina. While not all re-isolated sequences were identical to the original isolates, the sequence differences were fewer than what could be expected due to routine Sanger sequencing errors, confirming the findings from Poulsen and colleagues [19] and Andersen and colleagues [20] on Panamanian *Acromyrmex* species, in addition to suggesting that these patterns of colony-level specificity and long-term stability with *Pseudonocardia* exist across the genus *Acromyrmex*. Future work sampling workers from field colonies over several years would be desirable to confirm our findings in the presence of the potential selective pressure imposed by the horizontally transmitted pathogen [40] and the potential exposure to greater diversity of other strains of *Pseudonocardia*.

Our results, in combination with those of Andersen and colleagues (2013), support stable long-term maintenance of specific *Pseudonocardia* strains within colonies. However, these results could reflect stable associations of ant colonies with bacteria from the same phylogenetic clade, not a single specific strain. The reason for this is that 16 S is not a reliable marker to distinguish between closely related strains of bacteria (i.e., such a pattern could represent acquisition of a new strain of the same *Pseudonocardia* phylogenetic clade from another colony within the laboratory or even from environmental sources). Nevertheless, given the specific mechanisms involved in transmission identified in this study, it is likely that individual nests in fact do form stable associations with a single dominant *Pseudonocardia* strain. This is congruent with the work by Poulsen and colleagues (2005) and Caldera & Currie [41], which used *EF-Tu*, a more variable phylogenetic marker, and six house-keeping genes, respectively.

## Conclusions

The specific conditions for within-colony transmission described are expected to both ensure the efficient acquisition of the symbiont to new workers and to decrease the opportunity for other microbes to exploit this niche. This would benefit the ants by ensuring the association with their beneficial symbiont, and the symbiont by ensuring its transmission within the colony. This mode of transmission from older to newly eclosed adults is likely to be equivalent for the inoculation of virgin queens that are known to carry Actinobacterial exosymbiont on their cuticle during colony foundation [10], and would help ensure parent to offspring colony (vertical transmission) maintenance of the association between the maternal ant lineage (male reproductives do not carry *Pseudonocardia* and do not engage in colony founding) and the bacterial mutualist. This default vertical transmission mode, between host generations via virgin queens, should produce patterns of host-symbiont co-diversification, which to a large extent have been observed, only disrupted by events of horizontal transmission and acquisitions of free-living *Pseudonocardia* to the symbiosis [8], [41]. The identification of an apparently highly specific set of conditions for transmission of *Pseudonocardia* in *Acromyrmex* leaf-cutting ants has the potential to mediate long-term stability and host-symbiont specificity of the mutualism.

## Supporting Information

Figure S1 **Images of subcolony setup and ant colonization.** Experimental subcolony setup (a) with symbiotic (b, d, f, h, j) and aposymbiotic (c, e, g, i, k) ants. a) A subcolony with newly eclosed worker (lighter ant) and two major workers (darker ants), fungus garden and leaf fragments; images contrasting symbiotic (b, d, f, h, j) with aposymbiotic ants (c, e, g, i, k); c, d photos of adult ants, e-g dissecting microscope images of workers 14 days post-eclosion, h-k environmental scanning electron micrographs of workers 21 days post-eclosion. Note characteristic morphology of Actinobacteria in symbiotic ants and absence of these features in aposymbiotic ant. Photos b and c ©Alex Wild (used by permission), other images by Sarah Marsh.
(DOCX)

Table S1 **Experimental design overview.** The first column outlines each experiment, with subsequence columns providing the components in each subcolony: species of pupae (focal ant) and adult ants; number of exosymbiotic and aposymbiotic majors, and minors; fungus source colony ant species, and finally results (mortality and proportion of ants acquiring).
(DOCX)
