# Supplementary material for: Interaction between Workers during a Short Time Window Is Required for Bacterial Symbiont Transmission in Acromyrmex Leaf-Cutting Ants
Source: PLoS One. 2014 Jul 24;9(7):e103269. doi: 10.1371/journal.pone.0103269 (PMC4110003; doi:10.1371/journal.pone.0103269)
Supplement: Figure S1 — Images of subcolony setup and ant colonization. Experimental subcolony setup (a) with symbiotic (b, d, f, h, j) and aposymbiotic (c, e, g, i, k) ants. a) A subcolony with newly eclosed worker (lighter ant) and two major workers (darker ants), fungus garden and leaf fragments; images contrasting symbiotic (b, d, f, h, j) with aposymbiotic ants (c, e, g, i, k); c, d photos of adult ants, e-g dissecting microscope images of workers 14 days post-eclosion, h-k environmental scanning electron micrographs of workers 21 days post-eclosion. Note characteristic morphology of Actinobacteria in symbiotic ants and absence of these features in aposymbiotic ant. Photos b and c ©Alex Wild (used by permission), other images by Sarah Marsh. (DOCX) [file pone.0103269.s001.docx]

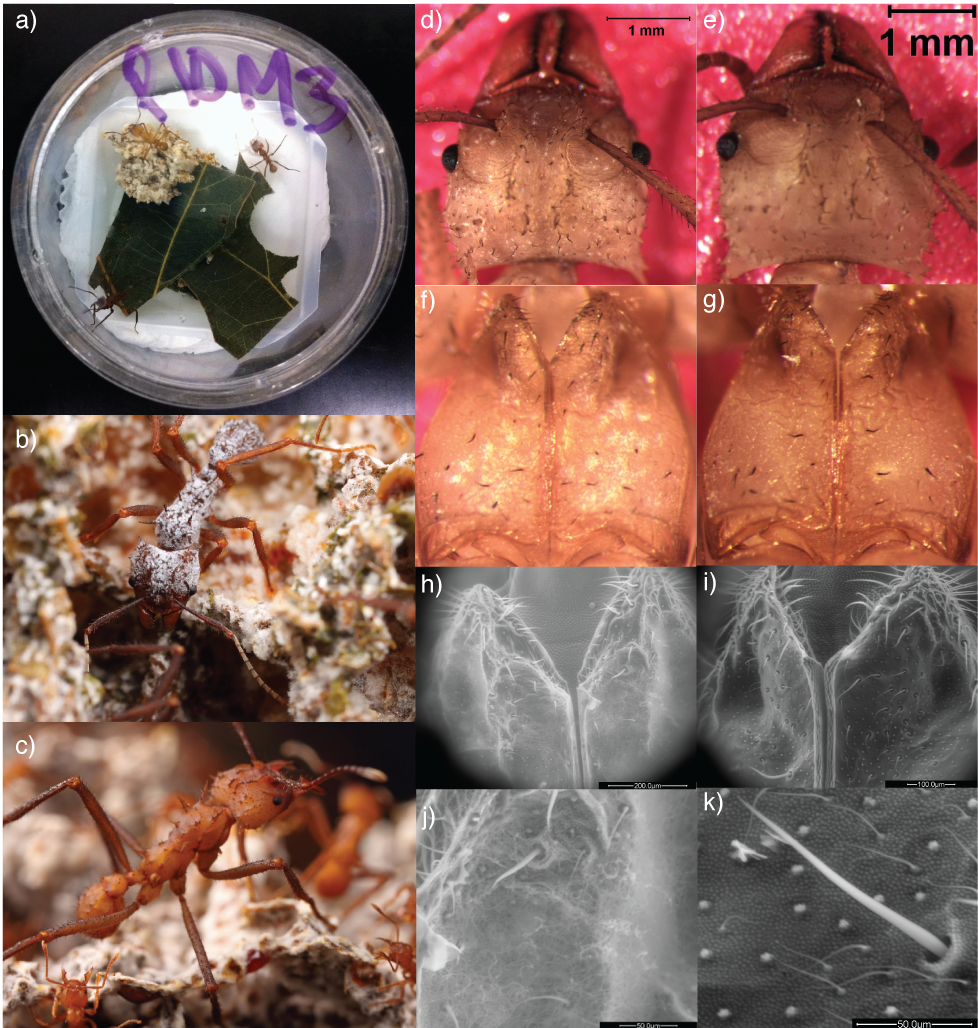


Figure S1. Images of subcolony setup and ant colonization.

Experimental subcolony setup (a) with symbiotic (b, d, f, h, j) and aposymbiotic (c, e, g, i, k) ants. a) A subcolony with newly eclosed worker (lighter ant) and two major workers (darker ants), fungus garden and leaf fragments; images contrasting symbiotic (b, d, f, h, j) with aposymbiotic ants (c, e, g, i, k); c, d photos of adult ants, e-g dissecting microscope images of workers 14 days post-eclosion, h-k environmental scanning electron micrographs of workers 21 days post-eclosion. Note characteristic morphology of Actinobacteria in symbiotic ants and absence of these features in aposymbiotic ant. Photos b and c ©Alex Wild (used by permission), other images by Sarah Marsh.
