# Supplementary material for: Interaction between Workers during a Short Time Window Is Required for Bacterial Symbiont Transmission in Acromyrmex Leaf-Cutting Ants
Source: PLoS One. 2014 Jul 24;9(7):e103269. doi: 10.1371/journal.pone.0103269 (PMC4110003; doi:10.1371/journal.pone.0103269)
Supplement: Table S1 — Experimental design overview. The first column outlines each experiment, with subsequence columns providing the components in each subcolony: species of pupae (focal ant) and adult ants; number of exosymbiotic and aposymbiotic majors, and minors; fungus source colony ant species, and finally results (mortality and proportion of ants acquiring). (DOCX) [file pone.0103269.s002.docx]

**Table S1. Experimental design overview.**

The first column outlines each experiment, with subsequence columns providing the components in each subcolony: species of pupae (focal ant) and adult ants; number of exosymbiotic and aposymbiotic majors, and minors; fungus source colony ant species, and finally results (mortality and proportion of ants acquiring).

| **Experiment** | **Pupa species** | **Adult species** | **# majors** | | **# minors** | **Fungus source** | **Survival to eclosion** | **# with exosymbiont** |
| --- | --- | --- | --- | --- | --- | --- | --- | --- |
|  |  |  | **Symbiotic** | **Aposymbiotic** |  |  |  |  |
| **Subcolony function** | *Acromyrmex echinatior* | *Acromyrmex echinatior* | 2 | 0 | 4 | *Acromyrmex echinatior* | 6/10 | 5/6 |
| **Fungus garden switch** | *Acromyrmex echinatior* | *Acromyrmex echinatior* | 2 | 0 | 4 | *Atta cephalotes* | 0/4 | 0 |
|  | *Acromyrmex octospinosus* | *Acromyrmex octospinosus* | 2 | 0 | 4 | *Atta cephalotes* | 7/12 | 7/7 |
|  | *Atta cephalotes* | *Atta cephalotes* | 0 | 2 | 4 | *Acromyrmex octospinosus* | 5/6 | 0/5 |
| ***Atta* ant cross-foster** | *Acromyrmex echinatior* | *Atta cephalotes* | 0 | 2 | 4 | *Acromyrmex echinatior* | 18/41 | 0/18 |
|  | *Acromyrmex octospinosus* | *Atta cephalotes* | 0 | 2 | 4 | *Acromyrmex octospinosus* | 14/20 | 0/14 |
| **minor workers** | *Acromyrmex echinatior* | *Acromyrmex echinatior* | 0 | 0 | 4 | *Acromyrmex echinatior* | 4/6 | 0/4 |
|  | *Acromyrmex echinatior* | *Acromyrmex echinatior* | thorax only | 0 | 4 | *Acromyrmex echinatior* | 8/11 | 0/8 |
|  | *Acromyrmex octospinosus* | *Acromyrmex octospinosus* | 0 | 0 | 4 | *Acromyrmex octospinosus* | 7/9 | 0/7 |
|  | *Acromyrmex octospinosus* | *Acromyrmex octospinosus* | thorax only | 0 | 4 | *Acromyrmex octospinosus* | 12/12 | 0/12 |
| **Major workers** | *Acromyrmex echinatior* | *Acromyrmex echinatior* | 4 | 0 | 0 | *Acromyrmex echinatior* | 5/8 | 5/5 |
|  | *Acromyrmex octospinosus* | *Acromyrmex octospinosus* | 4 | 0 | 0 | *Acromyrmex octospinosus* | 7/9 | 7/7 |
| **Major workers without exosymbiont** | *Acromyrmex echinatior* | *Acromyrmex echinatior* | 0 | 2 | 4 | *Acromyrmex echinatior* | 3/3 | 0/3 |
|  | *Acromyrmex octospinosus* | *Acromyrmex octospinosus* | 0 | 2 | 4 | *Acromyrmex octospinosus* | 7/9 | 0/7 |
